# Supplementary material for: Gauge your phage: benchmarking of bacteriophage identification tools in metagenomic sequencing data
Source: Microbiome. 2023 Apr 21;11:84. doi: 10.1186/s40168-023-01533-x (PMC10120246; doi:10.1186/s40168-023-01533-x)
Supplement: Supplementary file 2 — Additional file 1: Supplementary Fig. 1. Optimisation curves for tools resulting in score/probability thresholds. [file 40168_2023_1533_MOESM1_ESM.pdf]

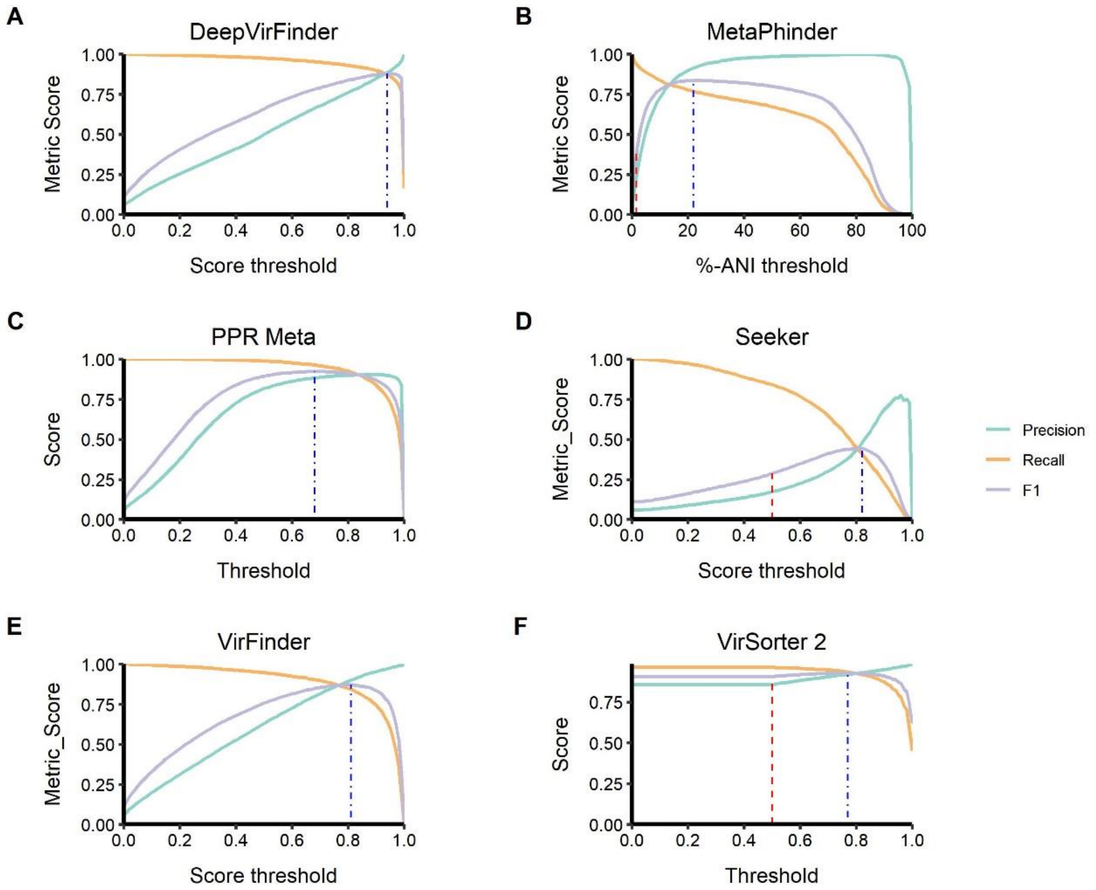

Supplementary Figure 1: Tool optimisation curves

Optimisation curves for tools that provide score/probability thresholds. Precision, recall, and F1-score are plotted against threshold. Red dotted lines highlight the performance at a default threshold where provided by the authors, and blue dotted lines indicate the threshold that has the highest F1-score for each tool. A. DeepVirFinder. Contigs that are predicted as viral with a score below or equal to the threshold are used in the metric calculation. B. MetaPhinder. Contigs with an ANI-% greater or equal to the threshold are used in the metric calculation. C. PPR Meta. Contigs with a score higher or equal to the threshold are used to calculate the metrics. D. Seeker. Contigs with a score higher or equal to the threshold are used to calculate the metrics. E. VirFinder. Contigs that are predicted as viral with a score below or equal to the threshold are used in the metric calculation. F. VirSorter2. Contigs with a score higher or equal to the threshold are used to calculate the metrics.
