# Supplementary material for: Gauge your phage: benchmarking of bacteriophage identification tools in metagenomic sequencing data
Source: Microbiome. 2023 Apr 21;11:84. doi: 10.1186/s40168-023-01533-x (PMC10120246; doi:10.1186/s40168-023-01533-x)
Supplement: Supplementary file 3 — Additional file 2: Supplementary Fig. 2. F1-score plots of tools that provide categorical thresholds. [file 40168_2023_1533_MOESM2_ESM.pdf]

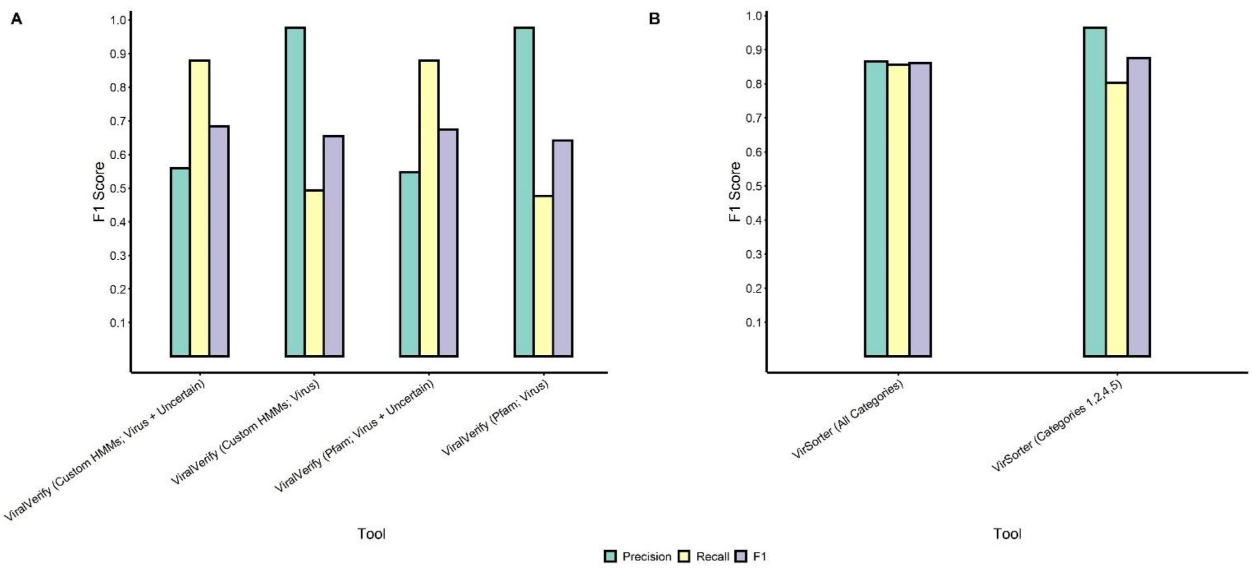

Supplementary Figure 2: Tool category optimisations

F1-score plots of tools that provide categorical thresholds. A. viralVerify has two thresholds, Virus, and Virus Uncertain. This was also combined with two databases: Pfam 34.0 HMM database, and the custom database provided on viralVerify's GitHub repository. B. VirSorter was run with two category sets, the first included all six categories, the second taking the highest two categories from its viral prediction (category 1 and 2) and its prophage prediction (category 4 and 5).
