# Supplementary material for: Gauge your phage: benchmarking of bacteriophage identification tools in metagenomic sequencing data
Source: Microbiome. 2023 Apr 21;11:84. doi: 10.1186/s40168-023-01533-x (PMC10120246; doi:10.1186/s40168-023-01533-x)
Supplement: Supplementary file 4 — Additional file 3: Supplementary Fig. 3. Comparison of artificial RefSeq contigs with reference genomes. [file 40168_2023_1533_MOESM3_ESM.pdf]

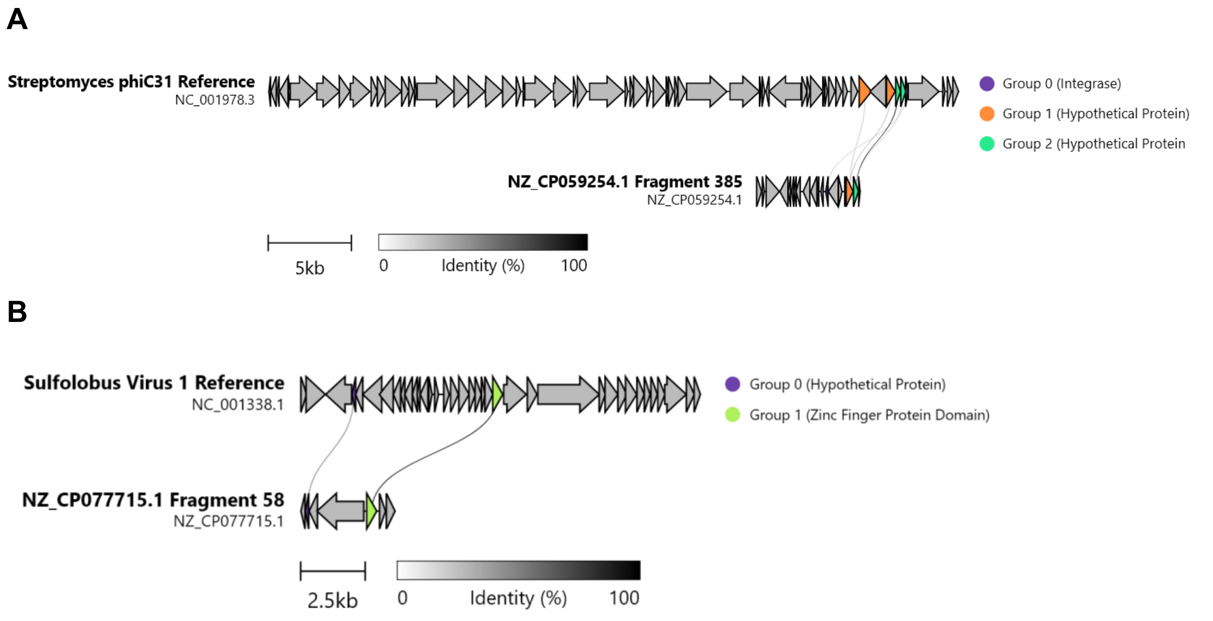

Supplementary Figure 3: Comparison of artificial RefSeq contigs with reference genomes.

Panel (A). Comparison of artificial RefSeq contig NZ_CP059254.1 fragment 385 to *Streptomyces* phiC31 phage reference genome (NC_001978.3). The 6217-bp contig NZ_CP059254.1 fragment 385 was identified as *Streptomyces* virus phiC31 by Kraken2. The contig originally belonged to the complete chromosomal sequence of *Streptomyces albidoflavus* strain J1074/R2. Panel (B). Comparison of artificial RefSeq contig NZ_CP077715.1 fragment 358 to *Sulfolobus* virus 1 reference genome (NC_001338.1). The 3647-bp contig NZ_CP077715.1 fragment 58 was identified as *Sulfolobus* virus 1 by Kraken2. The contig originally belonged to the complete chromosomal sequence of *Saccharolobus shibatae* strain BEU9. Contigs and reference genomes were annotated with pharokka and the genes were globally aligned and visualised with clinker. Homologous genes between the two sequences are coloured by homology and annotated with their function, as predicted by pharokka.
