# Supplementary material for: Gauge your phage: benchmarking of bacteriophage identification tools in metagenomic sequencing data
Source: Microbiome. 2023 Apr 21;11:84. doi: 10.1186/s40168-023-01533-x (PMC10120246; doi:10.1186/s40168-023-01533-x)
Supplement: Supplementary file 5 — Additional file 4: Supplementary Fig. 4. Upset plot of RefSeq phage artificial contigs predicted as viral by each tool. [file 40168_2023_1533_MOESM4_ESM.pdf]

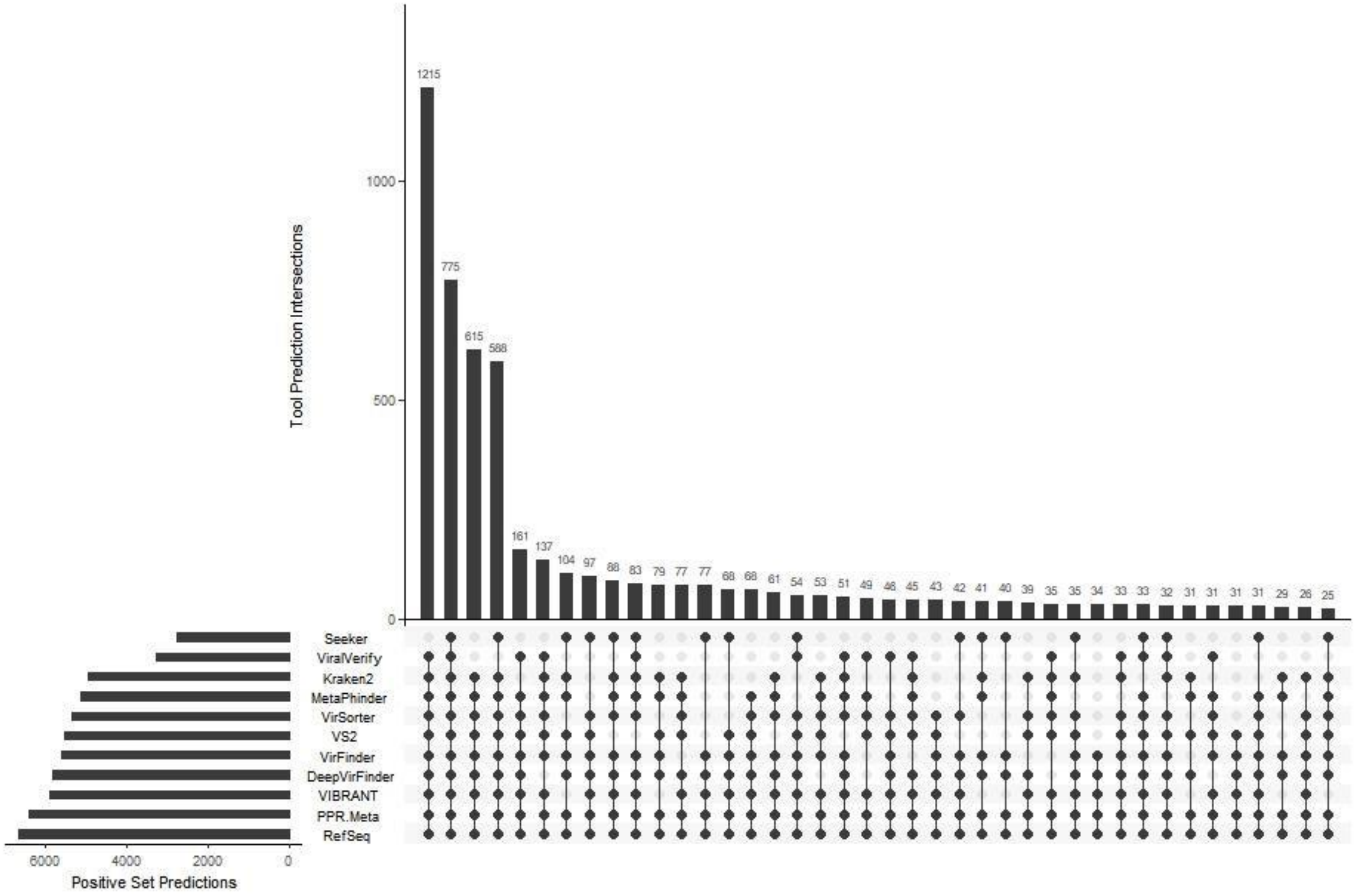

Supplementary Figure 4: Upset plot of RefSeq phage artificial contigs predicted as viral by each tool.

The bar chart on the left indicates the total number of artificial RefSeq phage contigs predicted as viral by each tool. The upper bar chart shows the intersection size between contigs that were predicted by each tool, with the dark connected dots on the panel below indicating which tools were included in the intersection. The top 50 intersections are shown.
