# Supplementary material for: Gauge your phage: benchmarking of bacteriophage identification tools in metagenomic sequencing data
Source: Microbiome. 2023 Apr 21;11:84. doi: 10.1186/s40168-023-01533-x (PMC10120246; doi:10.1186/s40168-023-01533-x)
Supplement: Supplementary file 6 — Additional file 5: Supplementary Fig. 5. Taxonomic analysis of each tool’s viral prediction on RefSeq phage fragments. [file 40168_2023_1533_MOESM5_ESM.pdf]

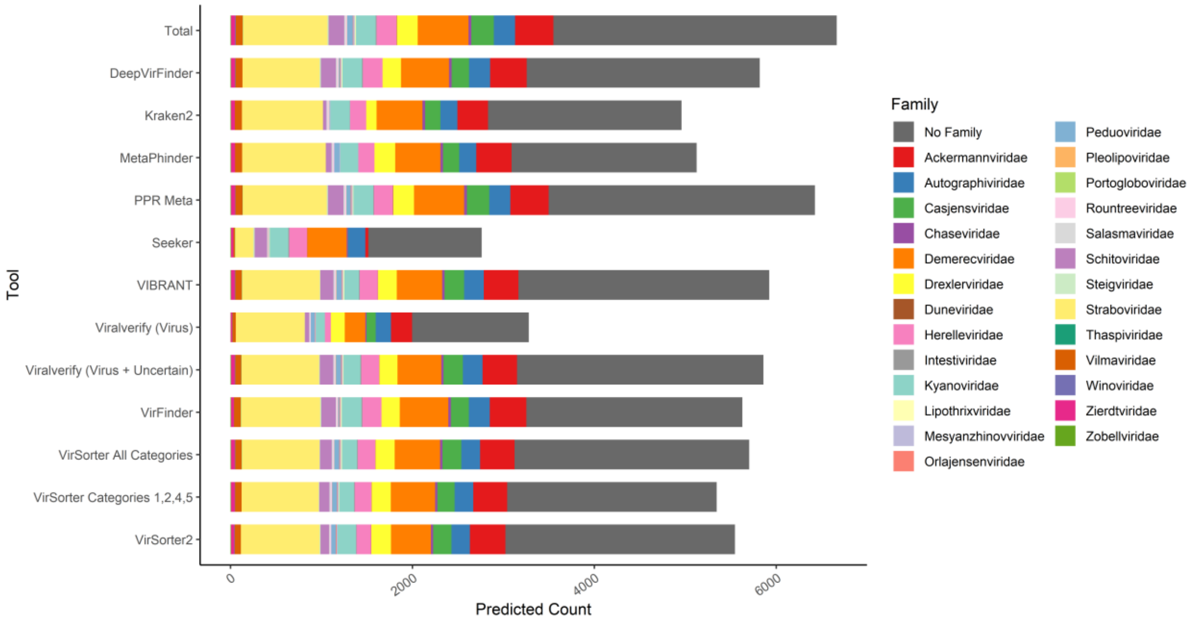

Supplementary Figure 5: Taxonomic analysis of each tool's viral prediction on RefSeq phage fragments.

Viral predictions of each tool on the RefSeq true positive phage genome fragments were computed and the associated RefSeq accessions were extracted. Taxonomy of these accessions were obtained using the R package Taxonomizr. Family level taxonomy was then visualised for each tool and compared to the taxonomy of the original fragments (shown in Total). Sequences with no family level taxonomy assigned were labelled as "No Family".
