# Supplementary material for: Gauge your phage: benchmarking of bacteriophage identification tools in metagenomic sequencing data
Source: Microbiome. 2023 Apr 21;11:84. doi: 10.1186/s40168-023-01533-x (PMC10120246; doi:10.1186/s40168-023-01533-x)
Supplement: Supplementary file 7 — Additional file 6: Supplementary Fig. 6. Taxonomy of false positive viral predictions on RefSeq chromosomal and plasmid fragments. [file 40168_2023_1533_MOESM6_ESM.pdf]

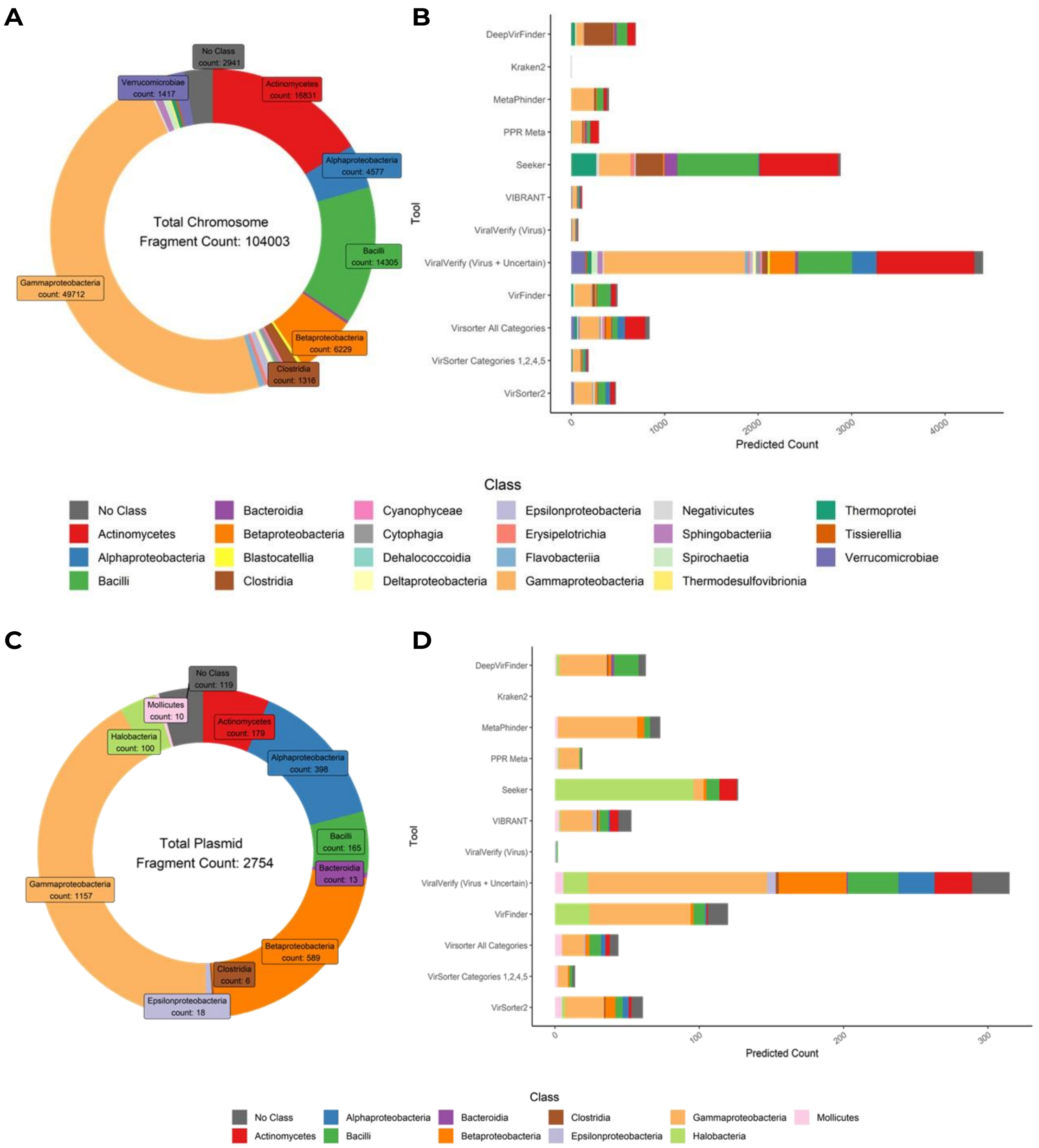

Supplementary Figure 6: Taxonomy of false positive viral predictions on RefSeq chromosomal and plasmid fragments.

Accessions of chromosomal and plasmid fragments were converted into class level taxonomy with Taxonomizr. Sequences with no class level annotation were labelled as “No Class”. Legends and colours are shared between parts A and B, and parts C and D. **A** – The taxonomic proportion of bacterial classes in the RefSeq chromosomal fragment dataset. Classes with over 1000 fragments were labelled for clarity. **B** – Taxonomy of false positive viral predictions on the RefSeq chromosomal dataset by each tool. **C –** The taxonomic proportion of bacterial classes in the RefSeq plasmid fragment dataset. All classes were labelled on the doughnut chart. **D** – Taxonomy of false positive viral predictions on the RefSeq plasmid dataset by each tool.
