# Supplementary material for: Gauge your phage: benchmarking of bacteriophage identification tools in metagenomic sequencing data
Source: Microbiome. 2023 Apr 21;11:84. doi: 10.1186/s40168-023-01533-x (PMC10120246; doi:10.1186/s40168-023-01533-x)
Supplement: Supplementary file 8 — Additional file 7: Supplementary Table 1. Bacteriophage strain composition of uneven mock community from Kleiner et al. [56]. [file 40168_2023_1533_MOESM7_ESM.pdf]

Supplementary Table 1: Bacteriophage strain composition of uneven mock community from Kleiner *et al.* [56]

Phage strain	Cell abundance (%)	Genome type	Host
ES18	0.363	dsDNA	*Salmonella enterica* serotype Typhimurium LT2
F0	2.925	dsDNA	*Salmonella enterica* serotype Typhimurium LT2
F2	0.250	ssRNA	*Escherichia coli* K12 with Flac+ Plasmid
M13	0.250	ssDNA	*Escherichia coli* K12 with Flac+ Plasmid
P22	21.250	dsDNA	*Salmonella enterica* serotype Typhimurium LT2

Composition, abundance, genome type and host of the five bacteriophage strains in the uneven mock community reported by Kleiner *et al.* [56].
