# Supplementary material for: Gauge your phage: benchmarking of bacteriophage identification tools in metagenomic sequencing data
Source: Microbiome. 2023 Apr 21;11:84. doi: 10.1186/s40168-023-01533-x (PMC10120246; doi:10.1186/s40168-023-01533-x)
Supplement: Supplementary file 9 — Additional file 8: Supplementary Table 2. Analysis of Similarity (ANOSIM) between non-metric multidimensional scaling of tools. [file 40168_2023_1533_MOESM8_ESM.pdf]

Supplementary Table 2: Analysis of Similarity (ANOSIM) between non-metric multidimensional scaling of tools

**Tool**	**ANOSIM statistic R**	**P-value**
DeepVirFinder	0.1264	0.1317
Kraken2	-0.07775	0.7357
MetaPhinder	-0.0442	0.6041
PPR Meta	-0.1073	0.8381
**Seeker**	**0.3872**	**0.0028**
VIBRANT	-0.1028	0.8298
VirFinder	0.002688	0.4552
VirSorter	-0.08822	0.7833
VirSorter2	-0.108	0.8375
viralVerify	0.02106	0.3985

ANOSIM of each tool against all other tools and the default. Significance values reported are not corrected for multiple comparisons. Values deemed to be significant (P < 0.05) after adjusting the alpha value with the Benjamini–Hochberg method are highlighted in bold.
